# *In vitro* simulation of drinking events in cattle^☆^^☆☆^

**DOI:** 10.1016/j.mex.2025.103593

**Published:** 2025-08-28

**Authors:** Md Shaheenur Rahman, Anna Chlingaryan, Peter C. Thomson, Mohammed Rafiq Islam, Angela M. Lees, Pablo Gregorini, Fabiellen Cristina Pereira, Cameron E.F. Clark

**Affiliations:** a Livestock Production and Welfare Group, Sydney Institute of Agriculture, School of Life and Environmental Sciences, The University of Sydney, Camden NSW 2570, Australia; b Department of Livestock Services, Krishi Khamar Sarak, Farmgate, Dhaka-1207, Bangladesh; c Sydney School of Veterinary Science, The University of Sydney, Camden NSW 2570, Australia; d School of Agriculture and Food Sustainability, Animal Science Group, The University of Queensland, Gatton QLD 4343, Australia; e Department of Agricultural Sciences, Lincoln University, Lincoln 7647, New Zealand; f Gulbali Institute for Agriculture, Water, and Environment. Charles Sturt University, 250 Boorooma St, North Wagga NSW 2650, Australia

**Keywords:** Reticulorumen temperature, Temperature dynamics, Reticulorumen ecosystem, Thermoregulation

## Abstract

Drinking causes a rapid decline in reticulorumen temperature (RT) followed by an exponential recovery, which may potentially impact the reticulorumen ecosystem. However, the nexus between drinking events and their effects on ruminal fermentation and microbial diversity has not yet been studied, either *in vitro* or *in vivo*. Although artificial (*in vitro*) rumen systems are widely used in ruminant research to simulate the reticulorumen environment, no such simulation has been described to consider the impact of drinking events on the reticulorumen environment. Therefore, we have developed a method for the *in vitro* simulation of drinking events in the fermentation jar where the jar temperature was considered a proxy for RT is reduced by adding a measured amount of cold water to the water bath, and the subsequent recovery period is achieved following a temperature profile regulated by a heating immersion circulator. This method enables the replication of RT fluctuations from drinking events, allowing for the monitoring of their impact on fermentation characteristics and microbial ecology in future research. The features of this method are:

Creation of a hypothetical drinking event

Estimation of volume and temperature of cold water for a drinking event

Establishing a temperature profile to regulate the recovery period

## Graphical abstract

**Figure:**
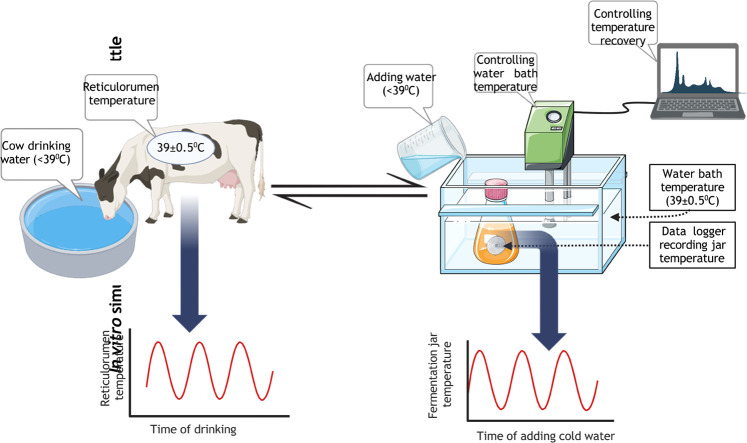

**Specifications table**

**Table:** 

**Subject area**	Agricultural and Biological Sciences
**More specific subject area**	*In vitro* study of the reticulorumen environment of cattle
**Name of your method**	*In vitro* simulation of drinking events in cattle
**Name and reference of original method**	*None*
**Resource availability**	Water bath (food quality stainless steel), Heating immersion circulator, Cold water, Temperature data logger, Computer, Fermentation jar (Glass jar, 250 ml)

## Background

The reticulorumen is a critical and complex microbial habitat that enables ruminant animals to efficiently digest complex carbohydrates, synthesise proteins, and convert fibrous plant materials into valuable nutrients and energy [1,2]. The temperature within the reticulorumen environment is tightly regulated between 38 °C and 42 °C, with an average temperature of 39 ± 0.5 °C, providing an optimal environment for the billions of microbes for their survival and proper functioning [[3], [4], [5], [6]]. Temperature deviations outside these normal ranges, either lower or higher, have been observed to decrease the adhesion of microbes to the rumen solids (fibrous substances) [7]. It has been reported in several studies that drinking can cause significant reticulorumen temperature (RT) fluctuations, ranging from 0.4 °C to 12.8 °C, with subsequent recovery periods of 15 min to 120 min [[8], [9], [10], [11], [12], [13]]. However, information regarding the impact of RT fluctuations due to drinking on feed digestibility, fermentation characteristics, and microbial diversity is limited. *In vitro* models of rumen fermentation have been developed to simulate the reticulorumen environment and have been extensively used in ruminant research, particularly in the fields of ruminant microbiology and nutrition. These models include continuous systems [14,15], which maintain a constant inflow of buffer and substrate along with a continuous outflow to mimic the steady-state conditions of the rumen; semi-continuous systems [16], which feature a continuous inflow of buffer, intermittent substrate feeding, and continuous outflow to simulate the periodic feeding patterns of ruminants; and batch systems [17,18], which have no inflow or outflow, with substrate and buffer added at the beginning and the system sealed for the duration of the experiment, making them suitable for short-term studies. These models have been instrumental in advancing our understanding of rumen fermentation dynamics over several decades. Among them, the Rumen Simulation Technique from a semi-continuous system and the ANKOM system from a batch fermentation system have been recommended to simulate rumen conditions and reticulorumen microbiome modelling [19]. However, simulating a reticulorumen environment, considering the effect of drinking events and their impact on microbial ecology, has not yet been studied in either of the *in vitro* systems. Compared to the semi-continuous system, the batch system is simple, easy to operate, relatively fast, and produces accurate results [19,20]. This system utilises fermentation jars that require minimal substrate and inoculum for fermentation and are placed in a water bath or equipped with a heating jacket to maintain a consistent temperature, *i.e.*, incubation temperature, of 39 ± 0.5 °C, simulating reticulorumen conditions [21,22]. The objective of this study was to develop an *in vitro* method to simulate drinking events in the fermentation jar and determine a protocol to establish the impact of RT declines and recovery periods. The establishment of such a protocol would allow for the impact of RT fluctuations due to drinking on feed digestibility, fermentation characteristics, and microbial diversity to be evaluated.

## Method details

An experiment was conducted using a water bath, cold water, and a fermentation jar at The University of Sydney’s Dairy Science laboratory (J.L. Shute Building, Camden campus, New South Wales, Australia), to create a drinking event and its associated temperature dynamics. The method of simulating drinking events with associated temperature drops and recovery periods was based on two basic thermodynamic principles: Richmann's Law of Mixtures [23] and Newton’s Law of Cooling/ Heating [24]. According to Richmann’s law, when two volumes of fluids at different initial temperatures are combined, they exchange heat until a thermal equilibrium is established [25]. On the other hand, Newton's Law of Cooling/Heating states that the rate of change of temperature of an object is directly proportional to the temperature difference between the object and its surroundings [26]. In cattle, RT is maintained approximately at 39.0 ± 0.5 °C. When cattle consume water below 39.0 °C, RT decreases as heat is exchanged between the water consumed and rumen content, then gradually returns to pre-drinking temperature over a certain time. In this study, RT dropping and its recovery were achieved in the fermentation jar by reducing the water bath temperature by adding cold water. These changes in temperature over time in the water bath and fermentation jar can be explained by Newton’s Law of Cooling/Heating. Simulating drinking events and their associated RT dynamics in an *in vitro* set-up was done as described below.

## Creation of a hypothetical drinking event

**Fig. 1:**
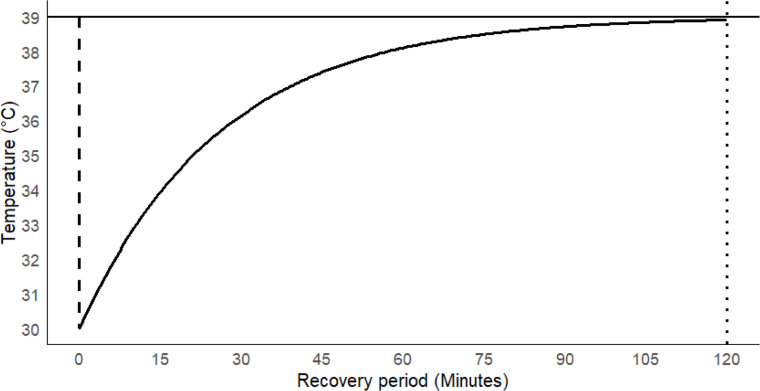
Hypothetical graph of a drinking event with the initial temperature drops of 9 °C (dashed vertical line) from the baseline temperature of 39 °C (solid horizontal line) and recovery period of 120 min (vertical dotted line).

Based on the concept of drinking and the thermodynamics of water, a hypothetical graph of a drinking event was created. This hypothetical drinking event considered a temperature drop of 9 °C from the baseline temperature of 39 °C and a recovery period (considered 99 % recovery of the dropped temperature) of 120 min, which aligned with the published literature mentioned earlier (Fig. 1).

## Equipment assembly

List of necessary materials and equipment for simulating drinking events:

• Food-grade stainless steel water bath (20 L)
• Heating immersion circulator
• Temperature data logger
• Data logger reader
• Cold water (≤4 °C)
• Plastic bucket
• Plastic tray
• Over sink rack
• Plastic mug/beaker
• Fermentation jar (250 mL)

## Assemble the heating immersion circulator with a water bath and run the device

The heating immersion circulator is designed for temperature control applications with liquid media in a water bath. This facilitates temperature control within the water bath using a predefined temperature regime. A commercially available CORIO CD heating immersion circulator (JULABO Technology, Germany https://www.julabo.com) with a working temperature range from +20 °C to +150 °C, temperature stability ± 0.03 °C, and temperature resolution 0.01 °C was used. A 20 L food-grade deep stainless-steel tray with dimensions of 53 cm × 32.5 cm × 15 cm (length × width × depth) was used as a water bath in this study. The heating circulator was fitted on the top edge of the water bath with a universal clamp, which was then connected to a socket and the computer with a provided cord and USB cable, respectively. According to the manufacturer's specifications and operating instructions, the pump and heater of the heating immersion circulator must always be covered entirely with bath fluid/water to operate this heating device. The current assembly requires 12.5 L of water to cover them. This assembly was used as media to simulate the drinking events with associated temperature drops and recovery periods in 250 mL fermentation jars (considered reticulorumen), generally used in the *in vitro* batch fermentation system.

## Placement of the fermentation jar and the temperature data logger

**Fig. 2:**
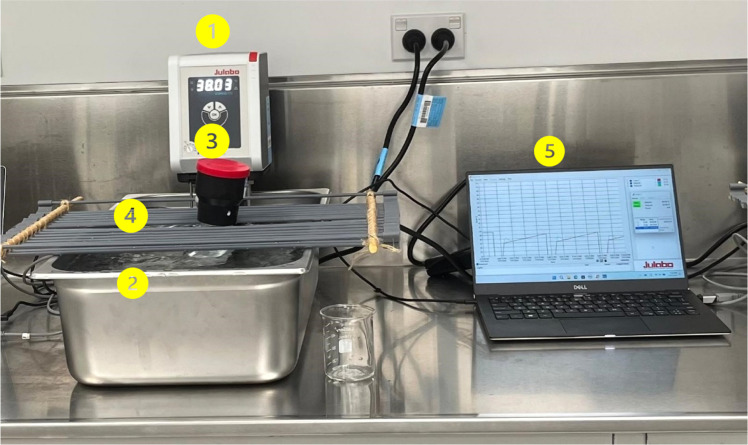
The assembly of a drinking event simulating unit consists of a heating immersion circulator**^1^** mounted on a stainless-steel water bath**^2^**, a fermentation jar sealed with an ANKOM module**^3^** holding a data logger inside, an over-sink rack**^4^** positioned across the water bath to hold the jar in place securely, and a data acquisition system comprising a laptop computer**^5^** connected to the circulator.

A temperature data logger was used to record the temperature inside the fermentation jar (iButton temperature logger, Thermochron eXtreme, range: −30 °C to 85 °C, resolution: 0.5 °C to 0.0625 °C, https://thermochron.com.au/). The activation time and the resolution of the data logger for temperature data logging were programmed using eTemperature software (https://etemperature.com.au) before placing it in the fermentation jar. After programming the data logger, it was placed into the fingertip of a disposable nitrile glove (Nitrile Gloves Large LC N338PF-l-LC MicroAnalytix Pty Ltd., Taren Point, NSW 2229, Australia) and tightened by making a knot for waterproofing. Then, it was placed inside the fermentation jar containing 100 mL of 39 °C water. The jars were then sealed with an ANKOM RF module and positioned in a water bath, held in place by an over-sink rack to prevent them from moving during simulating a drinking event by adding cold water to the water bath (Fig. 2).

## Estimating the volume and temperature of water required for creating a drinking event with a temperature drop of 9 °C

A drinking event is characterised by its associated temperature drop in RT and subsequent recovery period. For creating an *in vitro* drinking event to achieve a desired temperature drop of 9 °C, an estimate of water volume across temperatures is required. Richmann's law of mixtures was applied to estimate the desired temperature drops in the water bath and in the fermentation jars from the baseline temperature of 39 °C. Richmann's law calculates the final temperature of a mixture (*T_f_*) when two liquids of masses *m*_1_ and *m*_2_ at temperatures *T_1_* and *T_2_* are mixed, taking into account their respective heat capacity coefficients *c_1_* and *c_2_* represented as

(1) $$T_{f}=\frac{m_{1}c_{1}T_{1}+m_{2}c_{2}T_{2}}{m_{1}c_{1}+m_{2}c_{2}}$$

For example, mixing 1.0 L of water (*m*_1_) at 39.5 °C *(T_1_*) with 2.0 L of water (*m*_2_) at 4 °C (*T*_2_) results in the final temperature of the mixture (*T_f_*) at 15.8 °C. This is because of the mixture of two volumes of water whose specific heat capacity is 4.18 J/g °C.

**Fig. 3:**
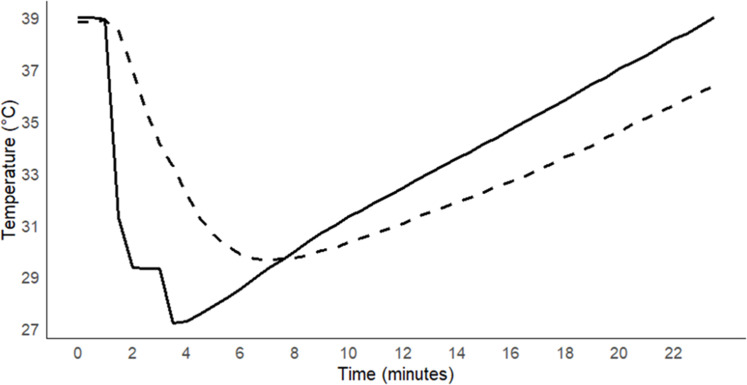
The observed temperature drops in the water bath (solid line) and the fermentation jar (dashed line) after adding cold water.

This method aimed to reduce the fermentation jar temperature by around 9 °C using cold water; however, the batch fermentation system does not allow any inflow or outflow within the jar. For this reason, cold water was introduced into the water bath to force a lower temperature in the fermentation jar. Despite the fermentation jar being situated within the water bath, the temperature decrease in the water bath from the baseline temperature (39 °C) does not equal the reduction observed in the fermentation jar (less than the drops in the water bath) (Fig. 3). This could be attributable to the density difference between water (1.0 g/cm^3^) and the fermentation jar (silica glass jar, 2.20 g/cm^3^) [27], as well as the heat transfer mechanism between two media. To resolve this issue, multiple simulation attempts have been conducted to determine the minimum temperature reduction in the water bath that will simultaneously generate around 9 °C decreases inside the fermentation jar. Considering the capacity of the water bath (20 L) used in our experiments, 4.0 °C water was used to achieve the desired temperature reduction. To determine the estimated temperature reduction in the water bath, Richmann's Law of Mixtures was applied. As outlined previously, 12.5 L (*m*_1_) of normal water must be added first to start the heating device, which regulates an increase in water bath temperature and maintains it at 39 °C (*T*_1_). The estimated temperature drops in the water bath from the estimated final temperature (*T*_f_) of the mixture (39 °C and 4 °C water) following the addition of different volumes of cold water (*m*_2_) at 4 °C (*T*_2_) are detailed in Table 1:

**Table 1:** Temperature drop in the water bath estimated by Richmann’s Law.

The volume of water *m_1_* (L)	Water bath temp *T*_1_ ( °C)	Sp. heat capacity of water *c*_1_ (J/g °C)	The volume of cold water *m*_2_ (L)	Temp of cold water *T*_2_ ( °C)	Sp. heat capacity of water *c*_2_ (J/g °C)	Final temperature in the water bath (*T*_f_) ( °C)	Temperature drops in the water bath (*T_1_*-*T_f_*) ( °C)
12.5	39.0	4.2	1	4	4.2	36.41	2.59
12.5	39.0	4.2	2	4	4.2	34.17	4.83
12.5	39.0	4.2	3	4	4.2	32.22	6.78
12.5	39.0	4.2	4	4	4.2	30.52	8.48
12.5	39.0	4.2	4.5	4	4.2	29.73	9.27
12.5	39.0	4.2	5	4	4.2	29.00	10.00
12.5	39.0	4.2	6	4	4.2	27.65	11.35
12.5	39.0	4.2	7	4	4.2	26.43	12.57

*m_1_ = The volume of water in the water bath, *T_1_ =* Baseline temperature in the water bath, *c_1_ =* Specific heat capacity of water, *m*_2_ = The volume of added cold water*, T*_2_ = Temp of the added cold water, *c*_2_ = Specific heat capacity of cold water, *T_f_* = Estimated final temperature in the water bath, *T_1_*-*T_f_ =* Estimated temperature drops in the water bath.

Following these estimated temperature drops in the water bath, several trials were undertaken with different volumes of water from 1.0 L to 7.0 L of 4 °C water to ensure that the desired temperature decline in the fermentation jar could be achieved. During these trials, data loggers were maintained inside the fermentation jar, per the description provided previously. It was found that the addition of 7.0 L of 4 °C water reduced the water bath temperature by approximately 12 °C while simultaneously reducing the fermentation jar temperature by around 9 °C (Fig. 3).

The water bath, having a capacity of 20 L, was initially filled with 12.5 L of water at 39 °C, to which 7.0 L of water at 4 °C was added in two steps to reduce the temperature of the water bath. Around 4.5 L of cold water was added to the water bath at the first step and waited until the water bath temperature reached equilibrium. At this point, the fermentation jar was temporarily transferred to a rectangular plastic tray, and subsequently, around 3–4 L of water from the water bath was shifted to this plastic tray to make room for adding the remaining cold water and maintaining the fermentation jar temperature during this transition time. Immediately after the water removal, the fermentation jar was returned to the water bath, and the remaining cold water was introduced. This stepwise addition of cold water slightly interrupted the decline in the water bath temperature (Fig. 3).

## Creating a recovery period for a drinking event

After reaching the RT to its lowest level, the time required to achieve within ± 0.10 °C of the pre-event level or 90 % of the dropped temperature is termed the recovery period [10]. After drinking, upon reaching the minima, RT increases exponentially toward the pre-drinking temperature. In this method, we used a software-programmed heating immersion circulator to regulate the recovery period from the minima to the initial baseline temperature in the water bath and fermentation jar (39 °C). Using this software, a temperature profile considering the initial rapid and gradual return of the water bath and fermentation jar temperature was created. This profile was set to achieve the recovery period in 2.0 h following the 9 °C drop in the fermentation jar (Table 2).

**Table 2:** Target water bath temperatures and the corresponding time intervals for achieving each temperature step.

Water bath Temperature to be reached at (deg C)	Time set for reaching the next step (hh:mm:ss)
27.00	00:00:00
36.00	00:30:00
38.00	00:30:00
39.00	01:00:00

*This table was created following the template outlined in JULABO EasyTemp software.

**Fig. 4:**
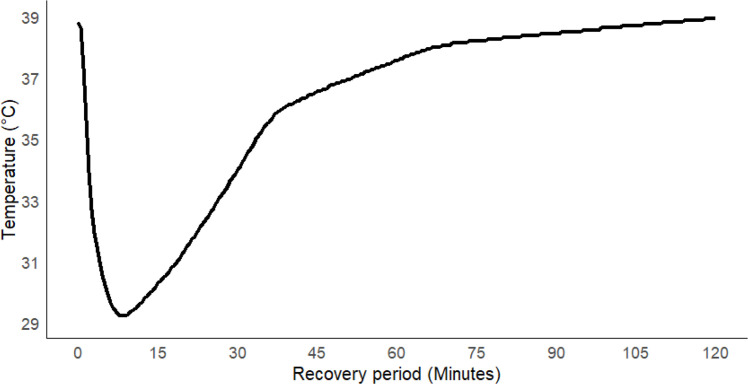
A single drinking event with a temperature drop of around 9 °C and a recovery period of about 120 min.

This temperature profile became activated when the water bath temperature reached 27 ± 0.01 °C due to the addition of 7.0 L of 4.0 °C water. Once the profile is activated, the water bath temperature is set to be reached at 36 °C from 27 °C in 30 min, then from 36.0 °C to 38.0 °C in another 30 min, and finally, it returned to the initial 39.0 °C from 38.0 °C in 1.0 hour. This completes a single drinking event episode with a 9.0 °C drop in the fermentation jar and a 120-minute recovery period (Fig. 4).

## Method validation

**Fig. 5:**
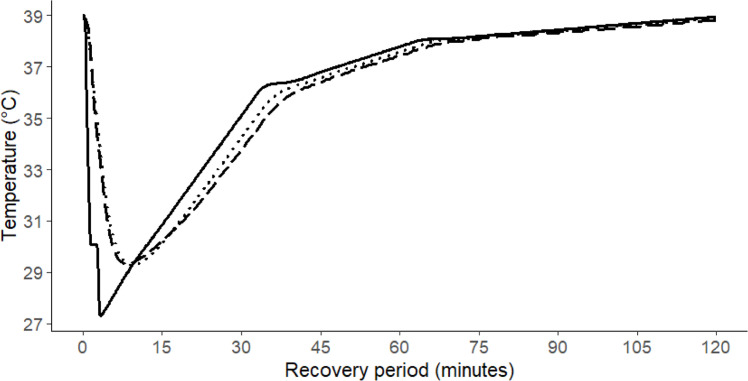
Comparison of observed (dashed line) and predicted (dotted line) jar temperatures based on water bath temperatures (solid line), as predicted by the heat transfer model.

Following the criteria of the hypothetical drinking event, an event with a temperature drop of around 9 °C and a subsequent recovery period of 120 mins was simulated in the fermentation jar. However, it was observed that it took several minutes (6–7 mins) for the jar temperature to be minimal (lagged response of the jar) compared to the time required for the hypothetical event and the event created in the water bath following the addition of cold water (Fig. 1; Fig. 3). Further, the temperature change in the jar across the recovery period was consistent with the temperature change in the water bath until reaching the pre-event level temperature at 39 °C (Fig. 5). To explore the association between changes in the bath temperature and changes in the jar temperature, Newton’s Law of Cooling/Heating was used. The water bath temperature was considered as input, and the fermentation jar temperature as output. Let *b*(*t*) be a function describing the temperature of the bath at time *t*, and let *j*(*t*) be the corresponding temperature of the jar within the bath at time *t*. Then according to Newton’s Law of Cooling/Heating, the rate of change of the jar temperature is proportional to the difference in temperature between the jar and the surrounding bath water, *i.e.*

(2) $$\frac{dj\left(t\right)}{dt}=-k_{j}\left[j\left(t\right)-b\left(t\right)\right]$$

where *k_j_* is a (positive) rate constant. Assuming the initial jar temperature, *j*(0), is *j*_0_, for an arbitrary input bath temperature function, *b*(*t*), the solution to this differential equation using the ‘integrating factor’ method is

(3) $$j\left(t\right)=\text{exp}\left(-k_{j}t\right)\left[j_{0}+k_{j}\int_{0}^{t}b\left(u\right)\exp\left(k_{j}u\right)du\right]$$

While the form of *b*(*t*) may be known in some situations, in the present study *b*(*t*) was under experimental control, so the above solution requires numerical methods. The bath temperature data set consisted of observations every 3 s, and to create a continuous function, linear interpolation was applied between consecutive time points using the ‘approxfun’ function in R, and numerical integration was undertaken using the integrate function in R.

The single parameter in the current model, *k_j_*, was estimated by least squares by choosing the value of *k_j_* to minimise

(4) $$\text{RSS}=\sum_{i=1}^{n}{\left(j_{i}-j\left(t_{i}\right)\right)}^{2}$$

where *j_i_* is the recorded (observed) jar temperature at time *t_i_, j*(*t_i_*) is the model-based predicted jar temperature, and *n* is the number of observations. The observed *versus* predicted temperature of the fermentation jar was assessed using the Root Mean Squared Error (RMSE), where

(5) $$\text{RMSE}=\sqrt{\text{RSS}\;/\;n}$$

For the data set displayed in Fig. 5, *k_j_* was estimated as 0.33, resulting in an RMSE of 0.22 °C.

It is anticipated that the magnitude of this temperature drop will be influenced by the volume and temperature of the cold water added to the water bath. Based on the maximum reticulorumen temperature drops following the drenching of cold water reported in published *in vivo* studies, we replicated this temperature drop of around 9 °C within the fermentation jars. This relatively small RMSE indicates the temperature time course in the jar follows what is predicted. This method effectively simulated a full drinking episode (Fig. 4). By adjusting the iteration number in the heating device software, multiple drinking events can be simulated using the protocol established here. Although our *in vitro* method is developed based on *in vivo* observations of reticulorumen temperature fluctuations following drinking, further validation under controlled *in vivo* experiments could help to address external and internal factors influencing RT drop and recovery and improve its applicability on a broader scale.

## Limitations

• Water baths <14 cm in depth are unsuitable for this method.
• If water is not sufficiently cold (>4 °C), the volume of water needs to be readjusted to achieve the desired temperature drop
• This is an indirect way of mimicking temperature fluctuations inside the fermentation jar, as no cold water is added to the jar
• This method is suitable for *in vitro* batch-type fermentation, so it incurs the limitation of batch fermentation

## Ethics statements

Not applicable

## Declaration of generative AI and AI-assisted technologies in the writing process

During the preparation of this manuscript, the author(s) did not use any AI and AI-assisted technologies.

## CRediT authorship contribution statement

**Md Shaheenur Rahman:** Methodology, Investigation, Validation, Writing – original draft, Data curation, Visualization. **Anna Chlingaryan:** Methodology, Supervision, Writing – review & editing. **Peter C. Thomson:** Software, Data curation, Visualization, Formal analysis, Writing – review & editing. **Mohammed Rafiq Islam:** Writing – review & editing, Methodology, Supervision. **Angela M. Lees:** Methodology, Writing – review & editing. **Pablo Gregorini:** Methodology, Writing – review & editing. **Fabiellen Cristina Pereira:** Methodology, Writing – review & editing. **Cameron E.F. Clark:** Conceptualization, Methodology, Funding acquisition, Project administration, Supervision, Writing – review & editing.

## Declaration of competing interest

The authors declare that they have no known competing financial interests or personal relationships that could have appeared to influence the work reported in this paper.

## Data Availability

Data will be made available on request.
